# New Formulation–Microporation Combination Approaches to Delivering Ciclopirox across Human Nails

**DOI:** 10.3390/pharmaceutics16010072

**Published:** 2024-01-04

**Authors:** Juliana Kishishita, Camila de Almeida Perez Pimenta, Danielle Patricia Cerqueira Macedo, M. Begoña Delgado-Charro, Leila Bastos Leal

**Affiliations:** 1 Departamento de Ciências Farmacêuticas, Núcleo de Desenvolvimento Farmacêutico e Cosmético (NUDFAC), Universidade Federal de Pernambuco (UFPE), Recife 50740-521, PE, Brazil; juliana_kishishita@hotmail.com (J.K.); camila.perez@ufpe.br (C.d.A.P.P.); leila.leal@nudfac.com.br (L.B.L.); 2 Departamento de Ciências Farmacêuticas, Laboratório de Análises Microbiológicas (LAM), Universidade Federal de Pernambuco (UFPE), Recife 50740-521, PE, Brazil; danielle.cerqueira@ufpe.br; 3 Department of Life Sciences, University of Bath, Claverton Down, Bath BA2 7AY, UK

**Keywords:** nail, ciclopirox, microporation, onychomycosis, Micolamina^®^, gel, thermogel, *Trichophyton rubrum*

## Abstract

Topical treatments for onychomycosis are of interest to those seeking to avoid systemic drug interactions and to improve systemic safety. This work aimed to develop aqueous-based, simple, and cost-effective vehicles that provide high solubility for ciclopirox and enable the delivery of an active through channels created by nail microporation. Following solubility tests, aqueous gels and thermogels based on hydroxypropylmethylcellulose and poloxamer 407, respectively, were loaded with 8% and 16% ciclopirox. Their performance was then compared to the marketed lacquer Micolamina^®^ in in vitro release tests with artificial membranes and in in vitro permeation tests with human nail clippings with and without poration. Finally, a microbiological assay compared the best gel formulations and the reference product. Little correlation was observed between the in vitro release and the permeation data, and the drug release was highly membrane-dependent. Ciclopirox nail retention in single-dose, porated nails tests was larger than in daily-dosing, non-porated nail conditions. The series of new gel and thermogel vehicles delivered ciclopirox more effectively than Micolamina^®^ in single-dose, porated nail experiments. The inhibition of *Trichophyton rubrum* activity was significantly increased with microporated nails when the gel formulations were applied but not with Micolamina^®^. Overall, the results suggest that the new vehicles could be successfully combined with nail microporation to improve the drug delivery and efficacy of topical antifungal medication while reducing the dosing frequency, facilitating patients’ adherence.

## 1. Introduction

Onychomycosis (ONC), the most common worldwide nail infection, is a fungal infection caused by both dermatophytes and non-dermatophytes [1,2]. Its incidence is rising due to the ageing population (ONC prevalence increases with age, affecting 20% to 50% of individuals over 60 years of age) [1,2,3,4,5] and increasing numbers of diabetic patients, who are 2.5 times more likely to develop ONC [2,5,6,7]. The challenges found in the development of efficacious topical treatments for onychomycosis have stimulated research on new formulations and enhancement approaches that may lead to more efficient topical medicines [8,9,10,11]. ONC topical treatments are time-consuming, requiring daily or weekly applications for up to one year [12], which leads to low compliance and treatment failure [13,14]. Ideally, new approaches should be developed that enable longer intervals between doses as to facilitate compliance.

Medicated lacquers including off-patent and more recent actives (ciclopirox, terbinafine, amorolfine, tavaborole, and efinaconazole) represent the topical medicines most sold globally for the treatment of ONC [15]. Most of these products are prepared with a blend of organic solvents that evaporate or quickly permeate the nail plate, so an impermeable hydrophobic polymeric film is formed on the nail surface [16]. Because this process is fast, whether drug delivery can take place from this film becomes crucial for a formulation to be efficient. Yet, it has been suggested that, as solvents disappear, they leave behind a residue of crystallized drug that is unable to partition into, or diffuse across, the nail plate [17,18]. In addition, because an antifungal must attain effective concentrations across the thickness (from 0.38 ± 0.05 mm to 0.63 ± 0.10 mm) of fingernails [19] and toenails (0.72 ± 0.20 mm) [20], nail poration [21,22] has been suggested as a tool with which to facilitate the penetration of drugs into the nail plate. However, the efficiency of this approach to deliver antifungals has been rarely explored [21]. In addition, for poration drug delivery to be effective, the approach requires formulations that flow through the pores without drying quickly and that avoid drug crystallization. Because of their quick metamorphosis, it is anticipated that solvent-based lacquers will not be a suitable choice for this application.

Nail lacquers containing ciclopirox are widely manufactured and prescribed globally, yet, as with most topical products for ONC, their clinical efficacy based on the cure rate is far from optimal [23,24,25]. The aim of this work was to develop aqueous-based, simple, and cost-effective vehicles, including permeation enhancers, that provide high solubility for CPO and are capable of delivering high fluxes of the drug, whether or not they are associated with microporation devices.

## 2. Materials and Methods

### 2.1. Chemicals

Ciclopirox olamine (CPO) batch 19D11-B023-0486 was purchased from Fagron (Rotterdam, The Netherlands). Micolamina^®^ lacquer batch T266 (CPO 8%, isopropyl alcohol, ethyl acetate, polymeth-acryliccopolyethylacrylate and dimethylsulfoxide) was purchased from a local distributor in Recife, Brazil. Isopropyl myristate, polyethylene glycol 400, Tween^®^ 20 (Polyoxy-ethylene sorbitan monolaurate), Span^®^ 80 (Sorbitan monooleate), Tween^®^ 80 (Polyox-yethylene sorbitan monooleate), ethyl alcohol, Kolliphor^®^ EL (Macrogolglycerol ricinoleate), Poloxamer 407, propylene glycol, isopropyl alcohol, potassium hydroxide, urea, hydroxypropylmethylcellulose (HPMC), sodium chloride, potassium chloride, sodium phosphate, potassium phosphate, sodium azide, oleic acid, BRIJ^®^20 (Polyoxy-ethylene 20 oleyl ether), sodium lauryl sulfate (SLS), and HPLC solvents methanol and acetonitrile from JT Baker, Phillipsburg, NJ, USA, were purchased from Casa do laboratório, Pernambuco, Brazil. Sabouraud dextrose agar and mycosel agar (PL1340) were purchased from Sigma-Aldrich (St. Louis, MO, USA) and Plastlabor (Rio de Janeiro, Brazil), respectively.

### 2.2. Nail Clippings Collection and Preparation for Tests

**Figure 1:**
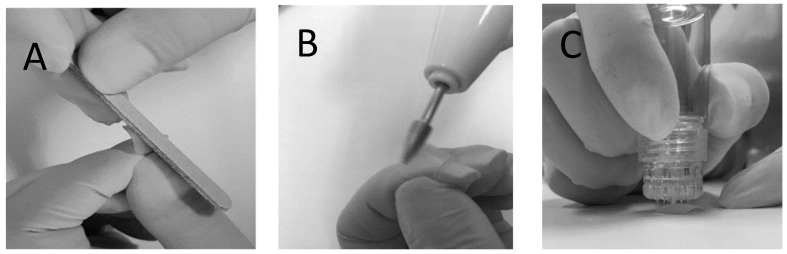
Nail sanding and microporation procedure. All nails underwent 2 h of hydration after which non-porated (NP) nails were manually sanded (Panel (**A**)) and microporated (M) nails were through electric filing (Panel (**B**)) and poration with a Hydra needle device (Panel (**C**)).

Human fingernail clippings at least 8 mm in length were donated by healthy volunteers after informed consent. Ethical approval was granted by the Human Ethics Committee of the Universidade Federal de Pernambuco (CAAE: 27554719.1.0005208). Following donation, the nail clippings were kept frozen at −20 °C until use. Before being used for in vitro permeation tests (IVPTs) and microbiological tests, the nail clippings were thawed and submitted to one of two physical treatments: The first nail treatment involved 2 h (pure water) hydration followed by sanding of the dorsal surface of the nails backwards and forwards 5 times using a nail file (Figure 1A); these nails are referred to as the non-porated (NP) nails henceforth. The second nail treatment involved 2 h of hydration followed by sanding of the dorsal surface of the nails using an electric nail file (Figure 1B), followed by microporating the nails 5 times using a commercially available device (Hydra.needle™, Guangzhou Ekai Electronic Technology Co., Ltd., Guangzhou, China) with titanium needles 0.60 mm in length (Figure 1C). The process resulted in approximately 20 pores being present in the diffusion area used for IVPT and microbiological tests. These nails are referred to as the microporated (MP) nails henceforth.

**Figure 2:**
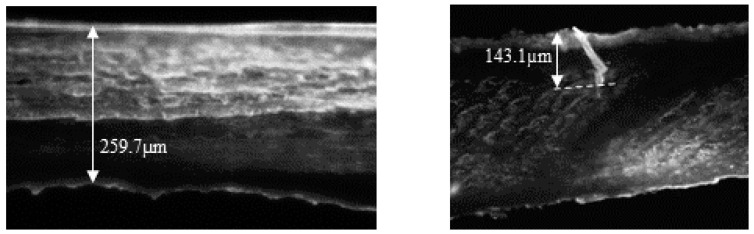
Microscopic transversal images: Left panel: a hydrated, filed, non-porated nail with a 259.7 µm thickness. Right panel: a hydrated, filed, and porated nail into which a 143.1 µm pore was created.

To assess the depth of the pores formed using the Hydra.needle™, three porated nails were cut in half and placed on a cryostat holder with the cut surface placed upwards. Blocking was performed in gel (Tissue-Tek^®^ O.C.T.™ Compound by Sakura) after which the samples were frozen and cut in a cryostat (Leica CM1860UV, Leica Mycrosystems, Wetzlar, Germany) at −27 °C. Five nail serial cross sections were obtained from each nail with a thickness of 7 µm and mounted on microscopy slides. During these sequential nail sections, one nail section was collected and placed on microscopy slides, whereas the next two were discarded. This cycle was repeated 5 times until 5 samples from each nail were collected for imaging. On the same day, the samples were observed under a fluorescence microscope (Leica DMI4000 B, Germany) using a 20x objective. The nails were photomicrographed and the thickness of the nails and the depth of the pores, when found, were measured. Data were evaluated using ImageJ software (National Institutes of Health, version 1.41 NIH). Figure 2 shows representative transversal cuts of a non-porated nail, following hydration and sanding only, and of a microporated nail following hydration, filing, and microporation. When pores were found, their depth was always less than the total thickness of the plate. For example, in the specimens shown in Figure 2, the total nail thickness was 259.7 µm, and the pore depth was 143.1 µm, suggesting that the microporation procedure did not create channels throughout the whole plate.

### 2.3. High Performance Liquid Chromatography (HPLC)

Samples were analyzed for CPO using an HPLC Nexera X2, Shimadzu^®^ (Kyoto, Japan), equipped with a diode array detector (DAD). The mobile phase combined acetonitrile (Pump A) and 1 mM EDTA aqueous solution containing 20 mM of phosphoric acid (Pump B) used in gradient mode. The cycle started with 10% of the organic phase (Pump A) and 90% of the aqueous phase (Pump B) as the initial condition, which was kept for 2.5 min, followed by 6.5 min comprising 60% Pump B and 40% Pump A, and by 1 min during which the column was rebalanced to the initial condition. A Gemini-NX C18 chromatographic column (150 × 4.6 mm 5 µm) was used, the flow rate was 1 mL/min, and the injection volume was 40 µL. CPO was detected at 303 nm. The total analysis time was 10 min, and the retention time for CPO was 6.2 min. The data were obtained using the LC Solution^®^ software version 1.25. The CLAE-DAD assay for CPO was partially validated following the guidelines of RDC No. 166/17 [26] and RDC No. 27/2012 [27] of the Brazilian National Health Surveillance Agency (ANVISA) for analytical and bioanalytical methods, respectively, with respect to the selectivity, linearity, accuracy, and precision of the method. The selectivity of the assay was verified using mobile phase, phosphate buffered saline (PBS) (pH = 7.4), PBS with sodium azide (30 mg/L), and human nail blank and nail extraction solution (70:30 ethanol/water), which was compared to a standard CPO solution (5 μg/mL) in the same solvent. Three samples of blank (untreated) nails from three different donors were cut into small pieces with scissors and placed in 1.5 mL microtubes containing 1 mL of ethanol/water (70:30), which was agitated using a shaking table (FinePCR^®^, Gunpo-si, South Korea) for 7 days after which the extracting solutions were filtered (0.45 μm) and quantified by HPLC-DAD. The linearity of the method was verified at concentrations of 5, 10, 20, 30, 50, and 100 μg/mL of CPO. Accuracy was determined by the repeatability test, in the same run (intraday) and in different runs on two different days (interdays) run by different analysts. In both cases, triplicate samples of three different concentrations (low, medium, and high) were used.

### 2.4. Solubility Study

The solubility of CPO in different vehicles was determined by adding excess drug (600 mg) to 1 mL to pure solvents, as well as to binary and ternary mixtures (Table 1) in a centrifuge microtube (Eppendorf^®^, Hamburg, Germany). The dispersions were homogenized by stirring at a controlled temperature (32 ± 2 °C), in a water bath (Quimis^®^, Diadema, Brazil) for a period of 72 h and then centrifuged (Eppendorf^®^, Germany) at 20,000× *g* rpm for 10 min. An aliquot was removed from the supernatant, filtered through a 0.45 µm PVDF membrane filter (Millex^®^, Darmstadt, Germany), and diluted with mobile phase (if necessary), after which the concentration of CPO was determined using the HPLC-DAD method described above.

### 2.5. Formulations

Based on the results from the solubility test, a series of gel (GH) and thermogel (GP) formulations were prepared according to the compositions described in Table 2. Based on the IVRT results obtained with these vehicles, some additional modifications on gel GH6 were tested (Table 3). To prepare gel formulations with HPMC, the polymer was added to ultrapure water, and the mixture was vortexed (Phoenix^®^, Garbsen, Germany) until the polymer was completely solubilized and a clear solution was formed. Subsequently, urea, potassium hydroxide, or SLS was added when relevant (Table 2 and Table 3). Isopropyl alcohol (IPA), propylene glycol (PG), or transcutol was added to the final total volume, and the drug was finally incorporated into the formulation using a magnetic stir bar. In the case of gels with Poloxamer^®^ (Darmstadt, Germany), the gelling agent was initially added to the water/isopropyl alcohol mixture. Subsequently, either PG or transcutol was added, and the preparation was vortexed and kept at a temperature of 22 °C for 24 h for complete solubilization of the polymer. Finally, the rest of the constituents and the drug were incorporated into the formulation using a magnetic stir bar. To assess the presence of crystals in the formulations chosen for the microbiological assay, 50 µL of the gels GH6 and GH6-GK were placed on glass slides at a temperature of 32 ± 1 °C and imaged in an optical microscope (Bioval, Curitiba, Brazil) after 10, 20, 30, 60, 90, 120, and 240 min (see representative images in the Supplementary Material, Section S2).

### 2.6. In Vitro Release Tests (IVRTs)

CPO release from the gel and thermogel formulations in Table 2 and Table 3 and from the marketed Micolamina^®^ was studied using vertical diffusion cells (PermeGear Inc. Bethlehem, PA, USA, diffusion area = 0.65 cm^2^). Donor formulations were 10 µL of 8% and 16% gel and thermogel formulations (corresponding to 1.23 mg/cm^2^ and 2.46 mg/cm^2^ of CPO respectively) and Micolamina^®^ (TheraSkin^®^ Farmacêutica, São Bernardo do Campo, Brazil). The receptor compartment was filled with 5 mL of phosphate buffered saline (PBS) (pH = 7.4). These series of experiments used three different membranes: a mixed cellulose esters membrane GSWP (0.22 µm pore size) from Merck Millipore Ltd. Millipore^®^ (Merck, Darmstadt, Germany), batch R5PA92282 (hydrophilic); a synthetic silicone elastomer membrane (Dow Corning^®^ 7-4107 (Corning, Corning, NY, USA)) (0.75 μm), and a hydrophobic PTFE membrane (0.45 µm pore size) from Filtrilo Ltd. (Colombo, Paraná, Brazil). The hydrophilic cellulose esters membranes were conditioned in phosphate buffered saline (PBS) for 12 h before use. The assembled system was maintained under magnetic stirring (300 rpm) (Fisatom 713d, Fisatom, São Paulo, Brazil) and kept at 32 ± 1 °C throughout the experiment. At 5, 10, and 20 min and at 0.5, 0.75, 1, 2, and 4 h, 0.5 mL were sampled from the receptor solution and replaced with fresh receptor medium. The samples were filtered (Millipore^®^ 0.45 µm) and analyzed by HPLC/DAD.

Comparisons among formulations were done by one-way ANOVA followed by Bonferroni’s multiple comparison tests. The level of statistical significance was established at *p* < 0.05.

### 2.7. In Vitro Permeation Tests (IVPTs)

IVPTs were performed using vertical diffusion cells with a nail adaptor (PermeGear Inc. Bethlehem, PA, USA, diffusion area = 0.196 cm^2^). The dorsal nail surface was placed facing the donor compartment, and the ventral surface was in contact with the receptor compartment, which was filled with 5 mL of PBS (pH = 7.4), ensuring sink conditions throughout the experiment.

The first type of IVPT experiment (MD-NP) involved multiple doses and filed/non-porated nails. In this case, 10 µL (equivalent to approximately 4.1 mg/cm^2^ and 8.2 mg/cm^2^ of CPO for formulations containing 8% and 16% of the active, respectively, were applied every 24 h. The surface of the nail was cleansed using two Johnson’s^®^ dry cotton swabs (Nova Brunswick, NJ, USA) between applying each new dose.

The second type of IVPT experiment (SD-MP) involved a single dose and filed/micro-porated nails. In this case, 50 µL (equivalent to approximately 20.41 mg/cm^2^ and 40.82 mg/cm^2^ of CPO) of formulations containing 8% and 16% CPO, respectively, were applied once at the beginning of the experiment.

In both experiments, the donor was occluded with Parafilm^®^ (Chicago, IL, USA); the cells were incubated at 32 ± 1 °C for 14 days, and the receptor was magnetically stirred (IKA^®^, Staufen, Germany). Subsequently, 0.5 mL of the receptor solution were sampled after 7 days (and replaced with fresh buffer) and at the end of the experiments (14 days). The samples were filtered (Millipore^®^ 0.45 µm) and analyzed using the previously described (HPLC-DAD) method.

**Figure 3:**
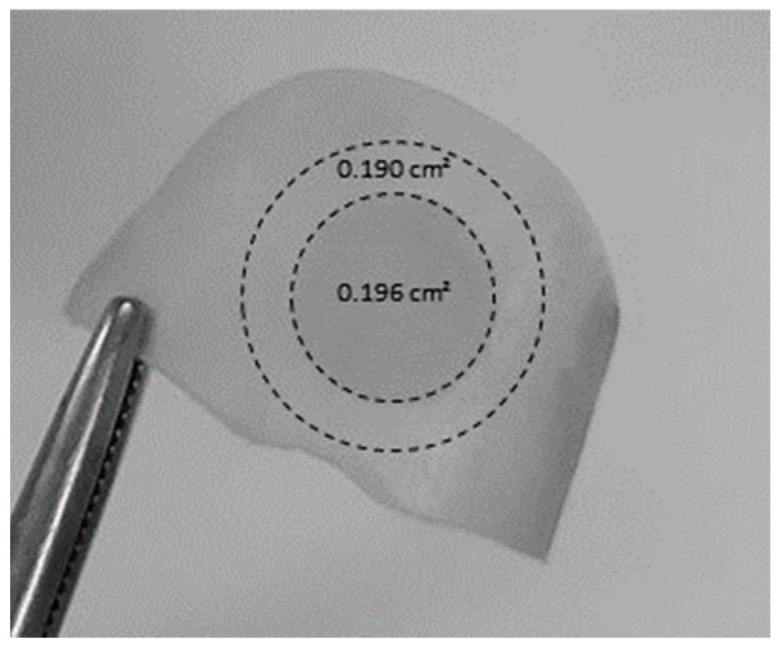
Image of a nail depicting the sections used to quantify CPO in the diffusional area (inner circle 0.196 cm^2^) and in the outer ring (0.190 cm^2^) at the end of IVPT experiments.

To evaluate the amount of drug present in the nail plate at the end of the experiments, the nail samples were removed from the nail adaptor and cleaned twice with isopropyl alcohol prep swabs (bio-Soma^®^, São Paulo, Brazil). The central circular region corresponding to the diffusional area (Figure 3) was cut out from the peripheral area and weighed, cut into small pieces, and placed in a centrifuge microtube (Eppendorf^®^, Germany) with extraction solution (1 mL of 70% ethanol). The centrifuge microtube was then shaken for 7 days, after which the solutions were filtered and analyzed using HPLC-UV as described above. An attempt was made to evaluate lateral diffusion, i.e., outside the diffusional area. For this, a ring (outer radius–inner radius ~2 mm) of nail specimen immediately outside the diffusion area was cut (Figure 3). The stability of CPO during the extraction procedure was previously established.

Comparisons among formulations were done by one-way and two-way ANOVA followed by Bonferroni’s multiple comparison tests and an unpaired Student *t*-test. The level of statistical significance was established at *p* < 0.05.

### 2.8. Microbiological Assay

**Figure 4:**
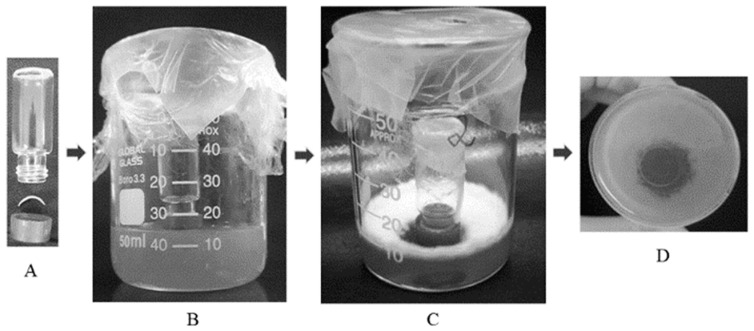
In vitro microbiological assay setup used in this work as adapted from Sleven et al. (2015) [28]. (**A**) Vial–nail ensemble before closure, (**B**) vial–nail ensemble placed on an agar medium during the preload step, (**C**) vial–nail ensemble placed on contaminated media, and (**D**) representative image of the inhibition halo formed.

The method described by Sleven et al. (2015) [28] with minor modifications as shown in Figure 4 was used. The bottom end of a 2 mL glass vial (Sigma-Aldrich^®^, EUA) was cut so that the vial could be used as a “donor chamber” for the formulations tested (see below). All material used was autoclaved at 121 °C for 15 min before mounting the system. Porated and non-porated nails were cleaned with isopropyl alcohol prep swabs (bio-Soma^®^, São Paulo, Brazil) and then sterilized for 30 min using an ultraviolet lamp (Veco, Campinas, Brazil). The following assembly procedure was carried out in a laminar flow hood: A nail clipping was mounted on the opening (5 mm diameter, 0.196 cm^2^) of the polypropylene cap of the vial with the nail dorsal side upwards; a rubber ring was placed on the ventral surface of the nail to prevent leakage, and the screw-on cap of the glass vial was then attached (Figure 4A). The ensemble was placed direct in contact with Sabouraud dextrose (SDA) agar, which simulated the nail bed (Figure 4B) located at the bottom of a 50 mL glass beaker and the set covered with Parafilm^®^.

For the preload step, a 50 μL single dose of the formulations was applied to the dorsal surface of the nails. After incubation for 14 days in an oven at 36.5 ± 1 °C, the vial–nail ensemble was transferred to a medium Sabouraud dextrose (SDA) agar contaminated with freshly inoculated *T. rubrum* (clinical isolate from internal collection provided by Sylvio Campos Medical Mycology Laboratory of the Biosciences Center—CB/UFPE, Recife, Brazil) as shown in Figure 4C and kept incubated in an oven at 36.5 ± 1 °C for another 7 days. Afterwards, whether an inhibition halo had been formed in the contaminated medium was verified (Figure 4D). The percentage inhibition was calculated as the area of inhibition halo relative to the total area of potential growth (11.94 cm^2^ in all cases). The area of the inhibition zone was estimated as that of a circle with the diameter measured with a digital caliper (0–150 mm). When an inhibition halo was formed, an additional step was carried to verify the watertightness of the setup. For this, the vial–nail ensemble was transferred at the end of the experiment to a new non-inoculated agar, and 400 μL of a 1% methylene blue aqueous solution were added to the donor chamber and observed for another 7 days [28]. If no inhibition halo formed, a direct mycological examination was performed on the nails [29,30]. If main fungal structures were not found in the microscopic evaluation, the fragments samples of this nail were cultivated on mycosel agar (selective medium for dermatophyte fungi) to check for potential *T. rubrum* growth.

Comparisons among formulations were done by two-way ANOVA followed by Bonferroni’s multiple comparison tests. The level of statistical significance was established at *p* < 0.05.

## 3. Results

### 3.1. Development of High Performance Liquid Chromatography (HPLC) for CPO

The N-hydroxylpyridone group in the CPO molecule interacts strongly with traces of metal ions in solvents and test systems and with adsorbents present in silica gel columns through a chelating effect. This results in a severe chromatographic tail and in a non-linear peak area versus concentration responses, making the direct determination of CPO challenging [31]. In fact, most published work on the quantification of CPO includes a derivatization step [32]. However, a direct method was preferred, so some analytical development work was conducted. First, a MS/MS detection method [33] was tried, but CPO could not be detected. Other methods have proposed including EDTA (another chelating agent) in the mobile phase to suppress the CPO’s chelating properties [34] and the addition of acetic acid [35] and phosphoric acid [36] to the mobile phase to improve the peak shape. Preliminary tests carried out with different concentrations of EDTA and different acids enabled the development of the method described above. The developed method exhibited selectivity, linearity, precision, and accuracy as specified in Resolution RDC No. 166 of 24 July 2017 [26]. CPO retention time was approximately 6.2 min. The limits of detection and quantification were 3.06 and 9.28 µg/mL, respectively.

### 3.2. Solubility Study

The solubility of CPO in different vehicles is presented in Table 1. Based on the results, pure solvents and binary, ternary, and quaternary mixtures were selected for the development of the gel and thermogel formulations.

### 3.3. Gel and Thermogel Formulations

CPO formulations were prepared with the composition shown in Table 2 and Table 3, and they remained macroscopically stable (without precipitates and/or color change as determined by simple visual inspection), provided drug stability (see Section S2 of the Supplementary Material), and were thus used for in vitro release (IVRTs) and permeation (IVPTs) tests. Formulations were prepared with 8% (*w/w*) CPO content, the same as the marketed product Micolamina^®,^ and with 16% CPO, as enabled by the drug solubility in the solvent mixtures (Table 1).

Following a first series of IVPT data with the formulations described in Table 2, additional formulations based of the GH6 gel were prepared (Table 3) and tested.

### 3.4. In Vitro Release Tests (IVRTs)

#### 3.4.1. Membrane Selection

Preliminary studies with three artificial membranes were performed, and the results are provided in the Supplementary Information. Figure S1 shows the CPO release from a gel (GH1), a thermogel (GP1) formulation, and Micolamina^®^, all containing 8% of the drug, and across three different artificial membranes for 4 h. PTFE and silicone membranes provided relatively consistent results: Micolamina^®^ released about 20% of the drug in 4 h, and the GH1 and GP1 formulations released less than 10% in all cases. On the contrary, GH1 and GP1 released more than 50% of their load in one just 1 h across the hydrophilic membranes, whereas only about 6% was released from the marketed product. Based on these results, the hydrophilic membrane was not used for further studies because of (a) the solvent back diffusion occurring with this membrane and (b) the membranes across which the marketed product released more of the drug, which was preferred for benchmarking purposes. All subsequent IVRTs involved PTFE and silicone membranes.

#### 3.4.2. IVRTs with Gel (HPMC) Formulations

**Figure 5:**
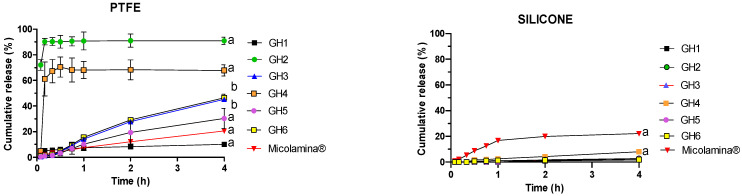
Mean (± SD, *n* = 6) CPO cumulative percentual release during IVRTs performed with HPMC gels GH1–GH6 formulations and Micolamina^®^ across PTFE membranes (**left** panel) and silicone membranes (**right** panel). Gels GH1, GH5, and Micolamina^®^ contained 8% CPO, whereas gels GH2, GH3, GH4, and GH6 contained 16% CPO. Significantly (*p* < 0.05) different percentages were released at 4 h: silicone membranes: (a) donor formulations were different from all others; PTFE membranes: (a) donor formulations were different from all others; (b) GH3 and GH6 were different from all formulations, but there were no differences found between them.

The CPO cumulative release (%) profile versus time (h) for HPMC gel formulations was different depending on the formulation and membrane used, whereas CPO release from Micolamina^®^ was relatively independent of the membrane used (Figure 5).

After the 4 h tests, the percentage of applied dose released across the silicone membranes by the gel products was less than 8.5% (range: 1.6–8.1%) in all cases and significantly (*p* < 0.01) less than the 22.15% released by Micolamina^®^. The total percentage of CPO released from GH4 was significantly (*p* < 0.01) larger than from the other gel formulations. Overall, these results suggest that the CPO release was at least partially rate-controlled by diffusion across the silicone membrane. In contrast, the CPO release across the PTFE membranes was more efficient and enabled the establishment of differences between the gel vehicles with percentage releases ranging between 10% and 91%. In this case, the drug release from the gels, with the exception of GH1, was more efficient than from Micolamina^®^ (~21%). Regardless of the type of membrane, no differences were found between the CPO total percentage released from GH3 and GH6. Finally, GH2 and GH4 provided a burst release of the drug across the PTFE membranes (Figure 5, left panel), which has been associated with poorer IVPT performance in previous work with tioconazole [21].

Given their better capacity to separate CPO release by different formulations, only PTFE membranes were used for IVRTs performed with the second series of GH6-based gel products (Table 3). The results are shown in Figure S2 of the Supplementary Data. In summary, none of these iterations improved the release of CPO significantly compared to the original GH6. In addition, CPO release was decreased for GH6-GL and GH6-GP donors compared to all others, perhaps due to their apparent increased viscosity.

#### 3.4.3. IVRTs with Thermogel (Poloxamer) Formulations

**Figure 6:**
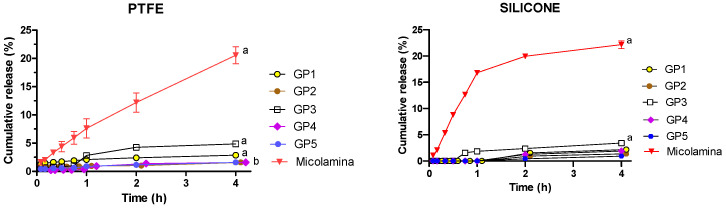
Mean (± SD, *n* = 6) CPO cumulative percentual release during IVRTs performed with thermogels GP1-GP5 and Micolamina^®^ across PTFE membranes (**left** panel) and silicone membranes (**right** panel). Left panel: To facilitate readability, data corresponding to GP2 have been nudged 0.1 h, and data corresponding to GP4 have been nudged 0.2 h. Thermogels GP2-GP5 were loaded with 16% of the drug, whereas Micolamina^®^ and GP1 contained 8% CPO. Significantly (*p* < 0.05) different percentages were released at 4 h: silicone membranes: (a) donor formulations were different from all others; PTFE membranes: (a) donor formulations were different from all others; (b) GP2, GP4 and GP5 were different from all formulations, but there were no differences found among them.

Figure 6 shows the CPO percentual release from poloxamer-based thermogels GP1-GP5 (Table 2) and Micolamina^®^ across PTFE and silicone membranes. Independently of the membrane used, the following observations were made: (a) The CPO percentage released from Micolamina^®^ was ~21% and significant larger (*p* < 0.01) than that of all thermogel formulations, (b) the percentage of CPO released from the poloxamer formulations was less than 5%, and (c) GP3, compared with the other thermogel donors, provided a significantly larger release. Finally, when the PTFE membrane was used, the thermogel GP1 containing 8% CPO released a significantly (*p* < 0.05) larger percentage of the dose than thermogels GP2, GP4, and GP5.

### 3.5. In Vitro Permeation Tests (IVPTs)

#### 3.5.1. Single Dose–Porated Nails (SD-MP) versus Multiple Dose–Non Porated (MD-NP) IVPTs

This first series of experiments comparing two IVPT designs, MD-NP and SD-MP, involved three formulations that contained 8% CPO: the gel GH1, the thermogel GP1, and the reference marketed as nail lacquer. The amount of CPO permeated to the receptor compartment (CPO-Rec) after 7 days was below the limit of detection in all cases. After 14 days, CPO-Rec was still undetectable in the MD-NP experiments, whereas, when poration was used, CPO-Rec (mean ± SD, *n* = 4) was 3.83 ± 0.55 µg/cm^2^ for GH1, 3.57 ± 0.36 µg/cm^2^ for GP1, and 1.40 ± 0.44 µg/cm^2^ for Micolamina^®^. CPO-Rec was significantly (*p* < 0.05) smaller when Micolamina^®^ was used compared with that of the gel and thermogel formulations.

**Figure 7:**
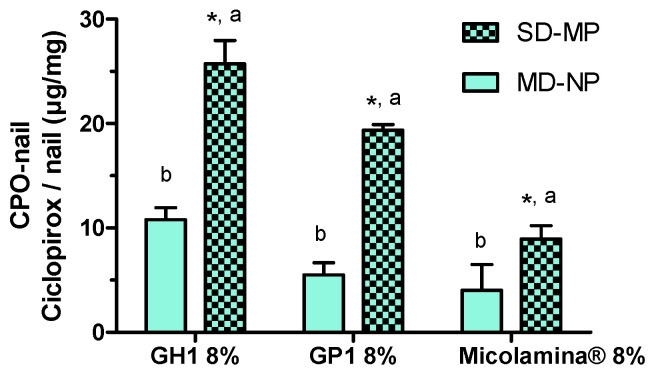
Mean (±SD, *n* = 4) CPO recovered from nails after IVPTs with gel (G8%), thermogel (TG8%), and Micolamina^®^ formulations. For all formulations, (*) poration significantly increased (*p* < 0.01) nail recovery. (a) Nail recovery was significantly (*p* < 0.001) different from that measured with all other formulations in the SD-MP tests; (b) nail recovery was significantly (*p* < 0.05) different from that measured with all other formulations in the MD-NP tests. Two-way ANOVA on factors “poration” and “formulation” followed by the Bonferroni post-test.

Figure 7 shows that poration significantly increased (two-way ANOVA, *p* < 0.0001) the amount of CPO recovered from the nail (CPO-Nail) (for all formulations). Independently of the nails being porated or not, the smallest CPO-Nail was found for Micolamina^®,^ and the largest was found for GH1. The amount of CPO found in the outside ring (Figure 3) was less than 5% of the amount found in the nail diffusional area in the MD-NP experiments and between 1.5% and 11% of the drug in the nail permeation area in the SD-MP experiments.

#### 3.5.2. Single Dose–Porated Nails (SD-MP) IVPTs

The above results suggested that combining nail microporation with a single fortnightly dose of an antifungal topical product could simultaneously deliver the active more efficiently and decrease the frequency of administration. Therefore, subsequent IVPT studies with gel and thermogel products took place in SD-MP conditions.

**Figure 8:**
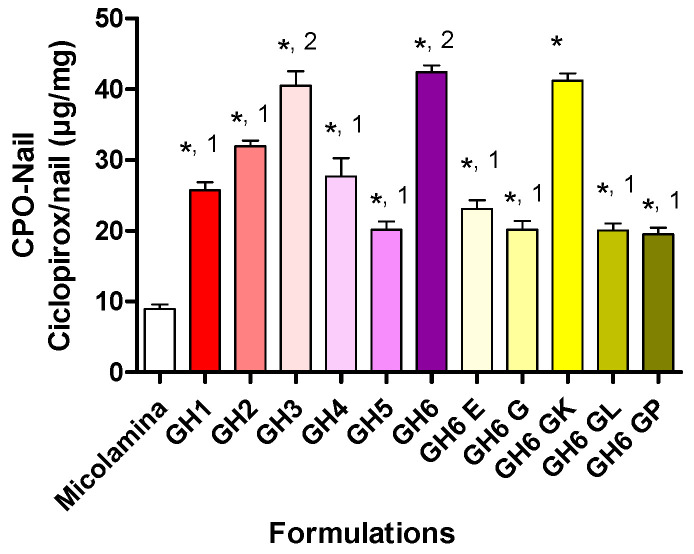
CPO amount recovered from the nail, CPO-Nail (mean ± SD, *n* = 4), following 14-day SD-MP experiments conducted with HPMC gel formulations and Micolamina^®^. Gels GH1 and GH5 and Micolamina^®^ contained 8% CPO, whereas all other formulations contained 16% CPO. (*) There was a significantly (*p* < 0.05) larger CPO recovery with Micolamina^®^. Label (1) and (2) identify groups of formulations providing similar CPO-Nail within the group but significantly (α < 0.05) different from formulations in the other group (one-way ANOVA, Bonferroni post-hoc test).

Figure 8 shows the results of SD-MP IVPTs conducted with HPMC-based gel formulations and with Micolamina^®^. Based on a one-way ANOVA and post-hoc Bonferroni test (*p* < 0.05). The amount of CPO recovered from the nail (CPO-Nail) was largest when the solvent combination included transcutol (GH6) or propylene glycol (GH3) in addition to water and isopropyl alcohol (GH2). CPO-Nail increased when the CPO concentration was increased in a formulation (GH1/GH2 and GH5/GH6). Finally, CPO-Nail was lower when KOH content was decreased (GH6-E and GH6-G versus GH6) or the formulation viscosity was increased (GH6-GL and GH6-GP versus GH6) and was not modified by an addition of ethyl acetate. Importantly, CPO-Nail was lower for Micolamina^®^ than for all 8% (GH1 and GH5) and 16% CPO gel formulations.

**Figure 9:**
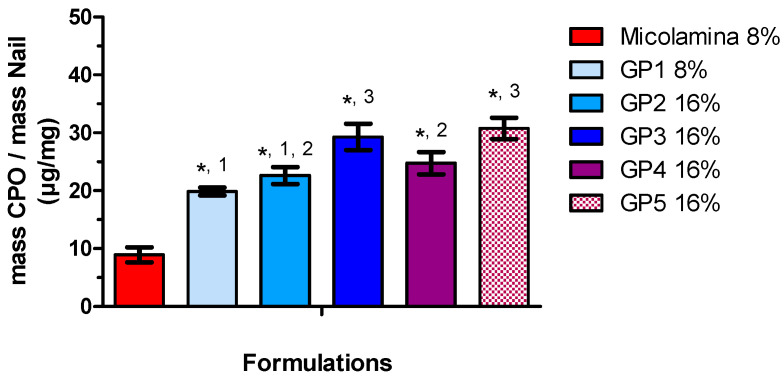
CPO amount recovered from the nail, CPO-Nail (mean ± SD, *n* = 4), following 14-day SD-MP experiments conducted with poloxamer thermogel (GP1-GP5) formulations and Micolamina^®^. Thermogels GP2-GP5 were loaded with 16% of the drug, whereas Micolamina^®^ and GP1 contained 8%. (*) There was a significantly (*p* < 0.05) larger CPO recovery with Micolamina^®^. Label (1), (2), and (3) identify groups of formulations providing a similar CPO-Nail within the group but significantly (α < 0.05) different from formulations in the other group (one-way ANOVA, Bonferroni post-hoc test).

Figure 9 shows the SD-MP IVPT results obtained with the thermogel vehicles. A one-way ANOVA followed by a post-hoc Bonferroni test indicated that CPO-Nail was significantly (*p* < 0.05) larger for all GP formulations than for Micolamina^®^. CPO-Nail was neither modified by CPO content (GP1/GP2) nor by the addition of transcutol (GP5) or SLS (GP4). The largest nail recovery was observed for gels containing 16% CPO containing KOH/urea and either a water/IPA/PG mixture (GP3) or water/IPA/transcutol (GP5).

Finally, a comparison between the two best-performing vehicles of each series, GH6 and GP5, indicated that CPO-Nail for the gel GH6 (42.42 ± 1.97 µg·cm^−2^) was significantly (*p* < 0.05, unpaired *t*-test) larger than the CPO-Nail for thermogel CPO-Nail (30.78 ± 1.83 µg·cm^−2^).

### 3.6. In Vitro Permeation (IVPTs) and Microbiological Tests with Selected Formulations

**Figure 10:**
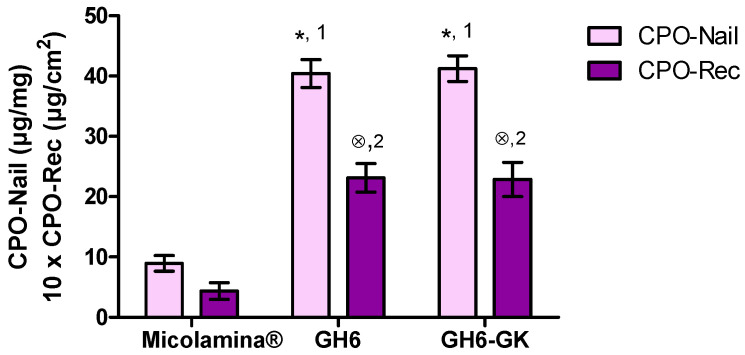
Mean (± SD, *n* = 4) CPO recovered from the nail (CPO-Nail) and permeated to the receptor compartment (CPO-Rec) after 14-day SD-MP experiments with selected 16% CPO gel formulations GH6 and GH6-GK and 8% Micolamina^®^. There was a significantly (*p* < 0.05) larger CPO-Nail (*) and CPO-Rec (⊗) compared with Micolamina^®^. Label (1) and (2) identify groups of formulations providing similar CPO-Nail and CPO-Rec within the group but significantly (α < 0.05) different from the corresponding metric for Micolamina^®^ (one-way ANOVA, Bonferroni post-hoc test).

The best performing formulations regarding CPO-Nail, GH6 and GH6-GK, were selected for further studies and comparison with Micolamina^®^. Figure 10 shows CPO recovery from the nail (CP-Nail) and delivered to the receptor (CPO-Rec) after 14-day SD-MP IVPTs. No CPO was detected in the receptor after 7 days of permeation in any case. After 14 days, CPO-Rec (mean ± SD, *n* = 4) for Micolamina^®^ (4.36 ± 1.38 µg/cm^2^) was significantly lower than that for GH6 (23.12 ± 2.34 µg/cm^2^) and for GH6-GK (22.86 ± 2.84 µg/cm^2^). Similarly, CPO-Nail (mean ± SD, *n* = 4) for Micolamina^®^ (8.93 ± 1.29 µg/mg) was significantly smaller (*p* < 0.05) than for GH6 (40.42 ± 2.32 µg/mg) and for GH6-GK (41.22 ± 2.12 µg/mg) for GH6-GK.

A final microbiological study was used to assess the effects of formulation (gel formulations GH6 and GH6-GK and Micolamina^®^) with the results shown in Table 4. Poration significantly increased (*p* < 0.001) the percentage inhibition of *T. rubrum* activity observed for both gel formulations but did not modify the inhibitory effect attained with Micolamina^®^.

## 4. Discussion

This work aimed to provide further insight on the potential of a microporation–formulation combination approach as a tool to improve the efficiency of topical therapies for onychomycosis. A proof-of-concept for this approach was provided in 2015 by Chiu et al. [22], who demonstrated the lateral diffusion of a marker across the nail plate from nanoparticle reservoirs immobilized in created pores; the nanoparticles were observed 70 µm deep into the nail. Further work combining microporation with tioconazole nanocapsules found that a single poration step enhanced delivery of the antifungal when combined with a single dose but not when followed by multiple doses of the nanocapsules [21]. Lastly, some work [37] testing dissolvable microneedle (circa 1000 µm depth × 500 µm middle dm × 700 µm external dm) array patches containing terbinafine and methylhydroxy-4-benzoate reported an extremely fast permeation across bovine hooves, comparable to that observed across a cellulosic filter membrane. However, the hooves had been soaked in 70% ethanol for 24 h prior to the studies, and the impact of this step on the poration effectiveness and permeation results is unclear. Prior work performed with human nail clippings [21,22] used a dermaroller (Infinite Beauty^®^) with 250 µm titanium needles, and given this “roller” poration action, trench-like structures or elongated cracks were created on the nail structure in addition to pores. Thus, a device (Figure 1) enabling a microporation action perpendicular with respect to the nail surface was used for this study, with the hope of creating channels that enter deeper into the nail structure. The Hydra.needle^TM^ device with 0.6 mm long titanium needles was operated manually, and an approximate number of pores (~20) was created in the IVPT diffusion area (0.196 cm^2^), which corresponds to ~102 pores/cm^2^. The surface area of fingernail and toenail plates ranges between ~1.5 and 5.3 cm^2^ [38,39], so to recreate an equivalent pore density in practical applications, a nail poration device would need to create 153–540 pores across the whole nail plate, respectively. The microscopic images of porated nails (Figure 2) suggest that the pores created were less than 150 µm deep, suggesting that less than half of the length of the titanium needles penetrated the nail plate. While poration was effective in this study, there is no information with which the results reported here can be compared. Overall, there is very little information regarding the optimum depth and density of nail poration required for the treatment of onychomycosis, so this is an area requiring further research. Importantly, an antifungal must reach effective concentrations across the whole nail plate as well as at the nail bed. This is in contrast with skin poration, which aims to bypass the stratum corneum barrier to deliver drugs to the live skin layers. A successful approach will need to consider pore density as well as how much a specific drug can diffuse laterally from the pores into the surrounding plate structure.

An important objective was to develop simple formulations with a high capacity to solubilize CPO, so the first stage of this project measured the solubility of CPO in a series of solvents and solvent mixtures. Based on this data (Table 1), a series of mixtures based on water/IPA, transcutol, and PG were selected as bases for the gel and thermogel formulations (Table 2 and Table 3). Because of the high solubility of CPO in the solvent mixtures selected, the new formulations could incorporate 16% CPO, doubling the drug content in the marketed product. Other additional components were based on prior reports [40] regarding their potential use in transungual delivery. The application sought in this work enabled flexibility in their formulation compared to traditional lacquers, which facilitated the attainment of this high solubility capacity. Organic solvents that evaporate fast have been preferred for the preparation of traditional medicated lacquers, so, once applied, they form rapidly dried films with a prolonged residence on the nail plate. However, as solvents evaporate, the drug may come out of the solution and crystallize, which stops further drug delivery. Indeed, when aqueous-based CPO lacquers have been prepared, their ability to maintain the drug in solution and to delivery it is highly increased compared to traditional organic lacquers, as recently demonstrated [41,42]. In the formulation–microporation approach, the new pores provide internal localization sites for the formulation; thus, adhesiveness properties of the formulation become secondary to their capacity to flow in and remain in the channels created. While the results attained in this work suggest that the formulations developed were able to enter the pores and to keep CPO solubilized for efficient delivery, further work is required to establish the formulation properties for optimum performance, among which viscosity, surface tension, and microfluidics seem the obvious starting point.

The next stage in this work explored the in vitro release of CPO across artificial membranes. IVRTs are an integral part of the quality testing of topical products, although there were no expectations that IVRT and IVPT performance will be correlated. However, IVPTs on nails are usually long (7–14 days), which significantly slows the screening of vehicles for further development. Thus, the availability of faster tests that identify best formulations for further characterization, even in the absence of quantitative correlations, would be advantageous. Previous work [21] performed with silicone membranes suggested that formulations that can extend the in vitro release of the drug provide better nail delivery compared to those showing a burst release profile. For this reason, short 4 h IVRTs were performed with PTFE and silicone membranes (Figure 5 and Figure 6), which demonstrated the important role of membrane choice. With regard to the commercial Micolamina^®^, very similar release profiles were obtained with both membranes, suggesting possibly that CPO release was primarily rate-limited by the lacquer in both experiments. In case of the gel formulations, CPO release seemed to be rate-controlled by the drug diffusion across the silicone membrane, as very little CPO reached the receptor in all cases. Similar behavior was observed for thermogel vehicles and silicone membranes. PTFE membranes enabled the differentiation of formulations, regarding both the total percentage released (10–91%) and the shape of the profile, with clear burst effects being observed for the GH2 and GH4 vehicles. Thus, it could be argued that CPO release across the PTFE membranes were primarily rate-limited by the drug release from the vehicles tested. Thermogel vehicles released little (1.6–5% in 4 h) CPO across the PTFE membranes, which, given the above discussion, could reflect an actual slower CPO release from the thermogel vehicles. These results underline how choosing an artificial membrane, the diffusion across which is not the rate-limiting process in the IVRT, is crucial for these tests to be meaningful.

Next, a first series of IVPTs aimed to assess the effect of poration on the amount of CPO present in the nail after 14-day IVPTs. Importantly, only one dose of the formulations was applied to the porated nails (SD-MP), whereas a daily dose was applied to the non-porated nails. (MD-NP). Despite the smaller (~a third) dose applied, CPO-Nail was significantly enhanced by poration in all cases. This is a significant finding, suggesting that formulation–poration combination approaches could simultaneously reduce the dosing frequency, facilitating adherence, reduce drug wastage, and improve antifungal delivery. Finally, the new formulations GH1 and GP1 provided a higher CPO-Nail than Micolamina^®^ in both the MD-NP and SD-MP conditions.

Following these positive results, all new vehicles were screened in SD-MP conditions using CPO-Nail as the metric for comparisons (Figure 8 and Figure 9). Overall, incorporating either transcutol or propylene glycol (GH3, GH6) to the water/IPA solvent system (GH2 resulted in better nail recovery, although this enhancing effect was not observed for the 8% CPO gels (GH1 and GH5). Doubling CPO content increased CPO-Nail proportionally for GH5 (20.1 ± 2.4 µg/mg) and GH6 (42.4 ± 2.0 µg/mg) but not for GH1 (25.7 ± 22.2 µg/mg) and GH2 (32.0 ± 1.5 µg/mg). All gel formulations, including those with 8% CPO, delivered more CPO to the nail than Micolamina^®^, which is not surprising, as the latter is formulated as a lacquer rather than a vehicle with the capacity to flow into the created channels. Unfortunately, no clear correlations were found between the IVRT results and CPO-Nail. For example, among the 16% CPO vehicles, the gels providing a burst release, GH2 and GH4, provided a smaller CPO-Nail than GH3, GH6, and GH6-GK but a similar or higher one compared to GH6-E, GH6-G, GH6-GL, and GH6-GP. Thermogels also provided higher CPO-Nail than Micolamina^®^ (Figure 9), and the highest nail recovery was observed also for vehicles (GP3 and GP5) containing transcutol and propylene glycol. Despite significant work on transungual drug delivery [36], predicting the enhancement that will be observed for a specific formulation and active pharmaceutical ingredient is still challenging. In addition, it is unclear how drug delivery from a film placed on top of a non-porated nail can be extrapolated to delivery from the formulation localized in the pores created in this work.

**Figure 11:**
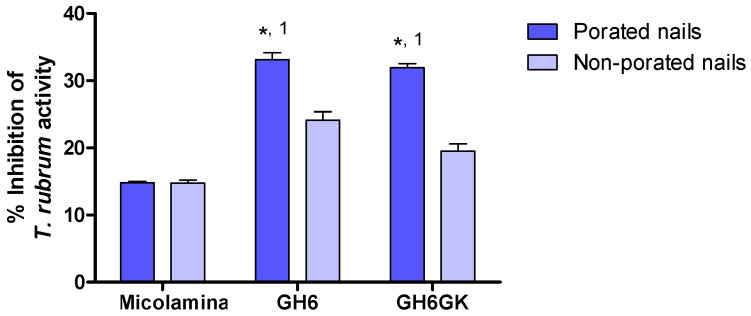
Mean (± SD, *n* = 4) percentage inhibition of *T. rubrum* activity after treatment of porated and non-porated nails with 16% CPO gels, GH6 and GH6GK, and 8% CPO Micolamina^®^. (*) There was a significant (*p* < 0.001) poration effect. Label 1 identifies two gels providing similar results within the group but significantly (*p* < 0.001) different inhibitory activity compared with Micolamina^®^. The three formulations provided different results (*p* < 0.01) when non-porated nails were used. Two-way ANOVA and post-hoc Bonferroni test.

Because gel vehicles provided a larger CPO-Nail than the thermogel vehicles, they were selected for subsequent IVPTs and microbiological tests. CPO-Nail and CPO-Rec were significantly higher for GH6 and GH6-GK than for Micolamina^®^ (Figure 10), with no differences being observed between the gels. Thus, in this work, the higher nail recovery observed for the gels was linked to a higher delivery to the receptor. This is important because, to treat onychomycosis effectively, a sufficient amount of the drug must be delivered across the nail plate (CPO-Nail) and must reach the nail bed (CPO-Rec). A limitation of the CPO-Nail metric is that it provides an average amount of drug across the nail, whereas information on the drug distribution across different layers of the plate would be more informative regarding therapy outcomes. Thus, to complete assessment of the new approach, a microbiological test based on a prior method [28] was performed using porated and non-porated nails (Table 4; Figure 11). In this test, the 14-day pre-load stage provided time for the drug to be released and diffused across the nail, so inhibitory activity could be seen when the nail was placed on the *T. rubrum*-contaminated medium, provided that sufficient delivery had taken place. These results further substantiate that the proposed formulation–microporation approach has the potential to improve the treatment of onychomycosis. The smaller but significant superiority observed for the gels with non-porated nails could be related to their capacity to keep the drug solubilized for a longer period of time or to their higher drug load. The larger superiority observed in the case of porated nails suggest that, in addition to these advantages, the gels were able to penetrate the channels and keep the drug solubilized to facilitate delivery from these sites. Poration did not improve the inhibitory effect of Micolamina^®^, probably because the organic-base lacquer dries before significant penetration into the pores can occur. Generally speaking, there was a very clear correlation between the results of the IVPTs (Figure 10) and the microbiological (Figure 11) tests, whether CPO-Nail or CPO-Rec were considered.

The results here indicate that formulation–poration combination approaches have a clear potential to improve the treatment of nail fungal infections. However, significant questions remain to be solved regarding the design of a nail poration device that adapts to the nail plate geometry and variability in its thickness and surface, the length and number of microneedles required, the best properties for a formulation to enter the pores and provide extended release, and finally the impact of poration on diseased nails.

## 5. Conclusions

This work provides evidence that formulation–microporation combination approaches may provide a superior alternative in the treatment of onychomycosis, delivering the drug more effectively and decreasing the frequency of administration from daily to fortnightly, potentially facilitating adherence. Significant questions that warrant further research on the approach remain.

## Figures and Tables

**Table 1:** CPO solubility (mean ± SD, *n* = 3) in a series of pure solvents and binary and ternary mixtures. Ultrapure Water was used in all cases. PG: propylene glycol; IPA: isopropyl alcohol. The solvent systems selected for formulation of the gel. Thermogel formulations are identified with an asterisk *.

Vehicle Composition (w/w)	Solubility (mg/mL)
IPA	195.93 ± 21.13
Water	23.40 ± 2.58
PG	283.80 ± 37.75
Isopropyl myristate	3.20 ± 0.51
Diethylene glycol monoethyl (DEGEE)–transcutol	130.42 ± 10.20
Tween 80	16.88 ± 0.27
Tween 20	21.03 ± 1.70
Cremophor EL	17.01 ± 5.34
Oleic acid	46.89 ± 10.48
Ethyl acetate	3.62 ± 0.24
Labrafac	4.06 ± 0.25
Water/IPA (50:50)	468.88 ± 31.00
* Water/IPA (30:70)	526.26 ± 54,00
Water/IPA (10:90)	429.42 ± 34.77
*Water/IPA/PG (33:33:33)	419.59 ± 79.40
Water/IPA/PG (25:50:25)	470.37 ± 25.11
Water/IPA/PG (15:70:15)	443.50 ± 46.85
* Water/IPA/transcutol (33:33:33)	379.71 ± 15.22
Water/IPA/transcutol (25:50:25)	382.63 ± 32.26
Water/IPA/transcutol (15:70:15)	420.64 ± 31.61
* Water/IPA/transcutol/ethyl acetate (16.65:33:33:16.65)	224.20 ± 34.08

**Table 2:** Composition of the gel (GH1-GH6) and thermogel (GP1-GP5) formulations. Ultrapure Water was used in all cases. HPMC: hydroxypropylmethyl cellulose; PG: propylene gycol; IPA: isopropyl alcohol.

Components (%, w/w)	Gel						Thermogel				
	GH1	GH2	GH3	GH4	GH5	GH6	GP1	GP2	GP3	GP4	GP5
CPO	8	16	16	16	8	16	8	16	16	16	16
HPMC	0.5	0.5	0.5	0.5	0.5	0.5					
Poloxamer 407							20	20	20	20	20
Water/IPA 30:70	89.5	81.5		83.5							
Water/IPA/PG 33:33:33			81.5				72	64	62	59	
Water/IPA/transcutol 33:33:33					89.5	81.5					62
Urea	1.0	1.0	1.0		1.0	1.0			1.0		1.0
Potassium hydroxide	1.0	1.0	1.0		1.0	1.0			1.0		1.0
SLS										5.0	

**Table 3:** Composition of the formulations GH6 and modified gel vehicles based on GH6. Ultrapure Water was used in all cases. HPMC: hydroxypropylmethyl cellulose; IPA: isopropyl alcohol; Lantette N: cetearyl alcohol/sodium cetearyl sulfate; Polawax NF: cetearyl alcohol/polysorbate 60; Span 80: sorbitan monooleate; Tween 80: polyoxyethylene sorbitan monooleate.

Components (%, w/w)	GH6	GH6-E	GH6-G	GH6-GK	GH6-GL	GH6-GP
CPO	16	16	16	16	16	16
HPMC	0.5	0.5	0.5	0.5	0.5	0.5
Water/IPA/transcutol 33:33:33	81.5					
Buffer/IPA/transcutol/ethyl acetate 16.65:33:33:16.65		82.4				
Water/IPA/transcutol/ethyl acetate 16.65:33:33:16.65			82.4	81.5	78.5	76.5
Lanette N					3	
Polawax NF						5
Span 80						20 drops
Tween 80					30 drops	
Urea	1.0	1.0	1.0	1.0	1.0	1.0
Potassium hydroxide	1.0	0.1	0.1	1.0	1.0	1.0

**Table 4:** Percentage of inhibition of *T. rubrum* activity observed with 16% CPO gel formulations GH6 and GH6-GK and with Micolamina^®^ using non-porated and porated nails (see Section 2).

Conditions	GH6	GH6-GK	Micolamina^®^
Porated nails	33.11 ± 2.14	31.91 ± 1.26	14.78 ± 0.46
Non-porated nails	24.14 ± 2.47	19.51 ± 2.16	14.75 ± 0.87

## Data Availability

The data presented in this study are available in this article and Supplementary Material.

## Supplementary Materials

The following supporting information can be downloaded at: https://www.mdpi.com/article/10.3390/pharmaceutics16010072/s1, Figure S1: CPO cumulative in vitro percentual release (mean ± SD, *n* = 6) from GH1, GP1, and Micolamina^®^ across cellulose, silicone, and PTFE membranes; Figure S2: CPO cumulative amount (mean ± SD, *n* = 6) released from the gels GH6, GH6-GK, GH6-GL, GH6-GP, GH6-E, and GH6-G across the PTFE membranes; Figure S3: Absence of crystals was made by placing 2 drops of each formulation on a slide, covered with a coverslip and then observed through an optical microscope; Figure S4: CPO formulations present the typical yellow colour (left panel) which may intensify 2–3 h after preparation (right panel); Table S1: Organoleptic characteristics, pH and CPO content (mean ± SD) of the formulations.
