# Supplementary material for: New Formulation–Microporation Combination Approaches to Delivering Ciclopirox across Human Nails
Source: Pharmaceutics. 2024 Jan 4;16(1):72. doi: 10.3390/pharmaceutics16010072 (PMC10819317; doi:10.3390/pharmaceutics16010072)
Supplement: Supplementary file 1 [file pharmaceutics-16-00072-s001.zip › pharmaceutics-2797859-supplementary.pdf]

## Supplementary material

# New formulation-microporation combined approaches to deliver ciclopirox across human nail.

**Juliana Kishishita[1], Camila de Almeida Perez Pimenta[1], Danielle Patricia Cerqueira Macêdo[2], M. Begoña Delgado-Charro[3,*] and Leila Bastos Leal[1]**

## S1. In vitro release test (IVRT) results

1.a Preliminary IVRT: Membrane selection:

Figures S1 show CPO release from a gel (GH1), a thermogel (GP1) formulation and from Micolamina®, all containing 8% of drug, and across 3 different artificial membranes for 4 hours.

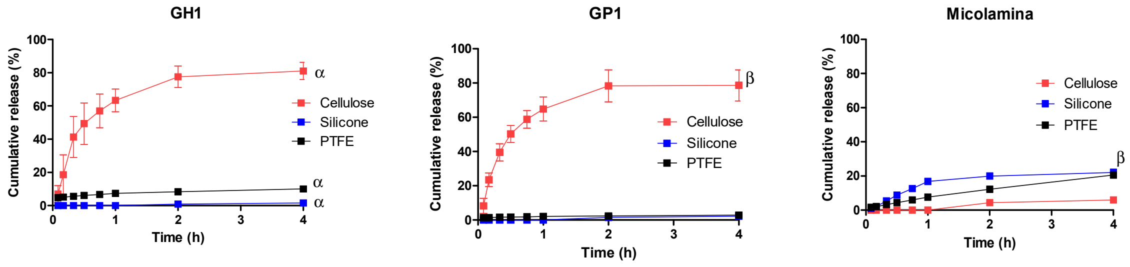


**Figure S1 –** CPO cumulative *in vitro* percentual release (mean ± SD, n=6) from GH1, GP1 and Micolamina® across cellulose, silicone and PTFE membranes. Results from one-way ANOVAs followed by Bonferroni's multiple comparison tests: $\alpha$: significantly ($p<0.05$) different percentage released across the three different membranes, $\beta$: significantly ($p<0.05$) different percentage released across cellulose and the other two membranes, but no differences found between silicone and PTFE membranes.

1.b IVRT results corresponding to the second series of gel products (Table 3) based on GH6 formulae.

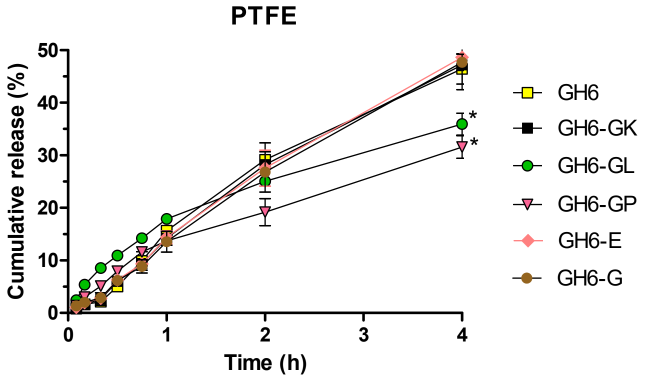


**Figure S2 -** CPO cumulative amount (mean ± SD, n=6) released from gels GH6, GH6-GK, GH6-GL, GH6-GP, GH6-E and GH6-G across PTFE membranes. GH6-GP and GH6GP (*) donors provided a significantly ($p<0.05$) smaller percentage released compared to all other gels but no differences were found between these two (one-way ANOVAs followed by Bonferroni's multiple comparison tests).

## S2. Additional formulation properties and drug stability.

Table S1 shows the organoleptic characteristics, pH and CPO content (mean ± SD) for the formulations. CPO content was determined immediately after preparation, and after 6 months storage at room temperature, and in all cases, the CPO loading was within the limit specified for topical formulations (90-110%).

Table S1 - Organoleptic characteristics, pH and CPO content (mean ± SD) of the formulations. * pH terrmined using pH indicator paper (MQuant®-Merck).

| Formulations | Consistency | Colour | Odor | pH | CPO content (%) T 0 | CPO content (%) T 6 months |
|---|---|---|---|---|---|---|
| **GH1** | Liquid | Colorless | Isopropanol residue | 12.5 ± 0.02 | 98.37 ± 0.07 | 97.11± 0.2 |
| **GH2** | Liquid | Slightly yellowish | Isopropanol residue | 12.4 ± 0.03 | 108.18 ± 0.025 | 99.23± 0.13 |
| **GH3** | Liquid | Slightly yellowish | Isopropanol residue | 12.7 ± 0.04 | 100.37 ± 0.027 | 96.73± 0.12 |
| **GH4** | Liquid | Colorless | Isopropanol residue | 10.8 ± 0.01 | 104.83 ± 0.02 | 99.45± 0.18 |
| **GH5** | Liquid | Colorless | Isopropanol residue | 12.3 ± 0.07 | 106.1 ± 0.015 | 96.66± 0.92 |
| **GH6** | Liquid | Slightly yellowish | Isopropanol residue | 12.1 ± 0.05 | 101.36 ± 0.036 | 95.99± 0.12 |
| **GH6-E** | Liquid | Colorless | Isopropanol residue | 10.6 ± 0.01 | 100.65 ± 0.078 | 98.02± 0.18 |
| **GH6-G** | Liquid | Colorless | Isopropanol residue | 10.9 ± 0.02 | 102.10 ± 0.068 | 99.82± 0.14 |
| **GH6-GK** | Liquid | Colorless | Isopropanol residue | 12.5 ± 0.03 | 99.75 ± 0.087 | 95.10± 0.15 |
| **GH6-GL** | Liquid | Colorless | Isopropanol residue | 12.8 ± 0.01 | 99.95 ± 0.058 | 98.77± 0.24 |
| **GH6-GP** | Liquid | Colorless | Isopropanol residue | 12.3 ± 0.02 | 102.02 ± 0.074 | 97.15± 0.27 |
| **GP1** | Slightly viscous liquid | Colorless | Isopropanol residue | 11.1 ± 0.01 | 103.45 ± 0.11 | 101.19± 0.12 |

| | | | | | | |
|---|---|---|---|---|---|---|
| **GP2** | Slightly viscous liquid | Slightly yellowish | Isopropanol residue | 10.8 ± 0.05 | 109.5 ± 0.17 | 96.84± 0.17 |
| **GP3** | Slightly viscous liquid | Slightly yellowish | Isopropanol residue | 12.3 ± 0.02 | 107.88 ± 0.035 | 104.00± 0.92 |
| **GP4** | Slightly viscous liquid | Slightly yellowish | Isopropanol residue | 10.5 ± 0.01 | 99.97 ± 0.33 | 96.02± 0.02 |
| **GP5** | Slightly viscous liquid | Slightly yellowish | Isopropanol residue | 12.9 ± 0.01 | 100.01 ± 0.04 | 95.00± 0.16 |
| **MICOLAMINA** | Liquid | Slightly yellowish | Organic solvent residue | 5* | 107.3 ± 0.04 | 98.34± 0.24 |

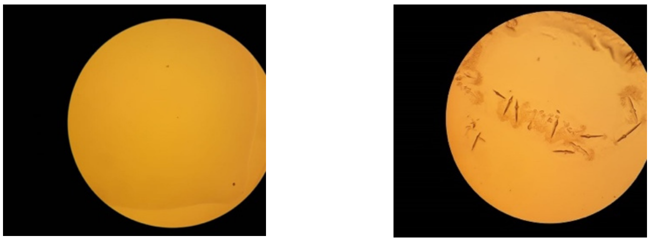

Top panel: Example of termogel formulation without crystals (left panel) and with crystals (right panel)

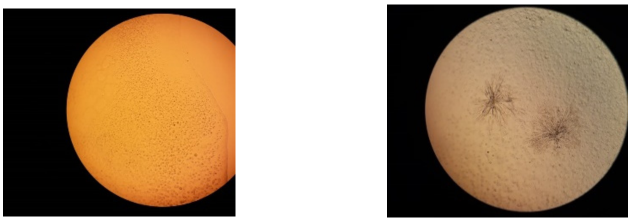

Bottom panel: Example of thermogel formulation without crystals (left panel) and with crystals (right panel)

**Figure S3 -** Absence of crystals was made by placing 2 drops of each formulation on a slide, covered with a coverslip and then observed through an optical microscope.

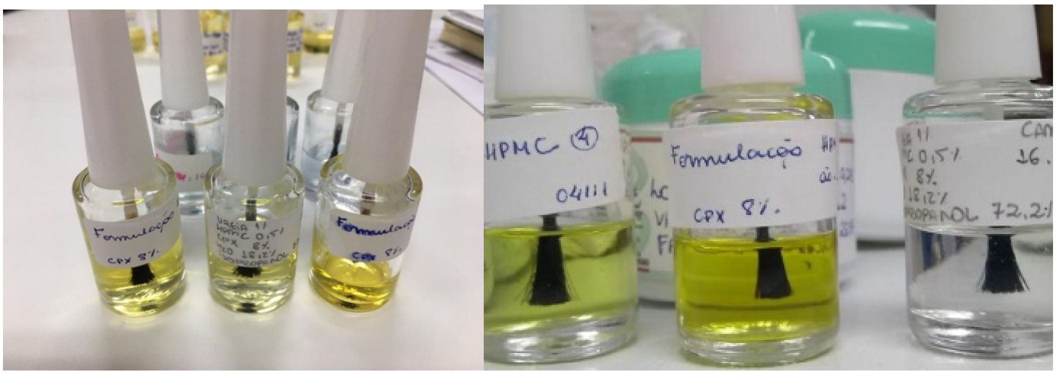


**Figure S4 –** CPO formulations present the typical yellow colour (left panel) which may instensify 2-3 hour after preparation (right panel).
